# Next-Level Remote Home Assessments With 3D Technology

**DOI:** 10.1093/geroni/igaf122.993

**Published:** 2025-12-31

**Authors:** Jaewon Kang, Mi Jung Lee, Consuelo Kreider, Kimberly Findley, Keith Myers, Sergio Romero

**Affiliations:** Colorado State University, Fort Collins, Colorado, United States; UTMB Health, The University of Texas Medical Branch, Galveston, Texas, United States; University of Florida College of Public Health and Health Professions, Gainesville, Florida, United States; Malcom Randall VA Medical Center, Gainesville, Florida, United States; Veterans Rural Health Resource Center-Gainesville (VRHRC-GNV), Gainesville, Florida, United States; Veterans Rural Health Resource Center-Gainesville (VRHRC-GNV), Gainesville, Florida, United States

## Abstract

Most older adults want to remain in their homes as they age. However, their homes may become unsafe or inaccessible, especially for those with disabilities or chronic conditions. Occupational therapists conduct home assessments to identify barriers that impact safety and accessibility for aging in place. Unfortunately, older adults, especially those in rural or remote areas, often face delays or are unable to receive these services due to logistic challenges posed by long distances and a shortage of trained clinicians. To improve access to home assessment services, videoconferencing has been recently adopted in practice. However, clinicians often report challenges such as unstable internet connections, limited field of view, difficulty measuring home environments, and heavy reliance on older adults and their care partners, which impact the quality of assessments. Our project tested 3D modeling technology, commonly used in real estate, to overcome these limitations. Occupational therapists in the study reported that 3D models allowed them to independently observe home environments from multiple perspectives, including 360-degree views, bird’s-eye views, and floor plans, while also enabling accurate remote measurements. By reducing reliance on high-speed internet and care partners, clinicians can conduct more comprehensive assessments anytime and anywhere, providing older adults with better access to essential services for aging in place.

